# Dairy *Propionibacterium freudenreichii* ameliorates acute colitis by stimulating MUC2 expression in intestinal goblet cell in a DSS-induced colitis rat model

**DOI:** 10.1038/s41598-020-62497-8

**Published:** 2020-03-26

**Authors:** Seongho Ma, Jiah Yeom, Young-Hee Lim

**Affiliations:** 1 Department of Integrated Biomedical and Life Sciences, Graduate School, Korea University, Seoul, 02841 Republic of Korea; 2 Department of Public Health Science (Brain Korea 21 PLUS program), Graduate School, Korea University, Seoul, 02841 Republic of Korea; 3 Department of Laboratory Medicine, Korea University Guro Hospital, Seoul, 08308 Republic of Korea

**Keywords:** Physiology, Gastroenterology

## Abstract

An intact mucus layer is important in managing inflammatory bowel disease (IBD). Dairy *Propionibacterium freudenreichii* has probiotic potential, produces propionic acid and is known to promote health. The aim of this study was to evaluate the effects of *P. freudenreichii* on the improvement of colitis. LS 174T goblet cells and a dextran sodium sulfate (DSS)-induced colitis rat model were used to investigate the *P. freudenreichii*-induced stimulation of mucin production *in vitro* and *in vivo*, respectively. The mRNA and protein expression levels of MUC2, a main component of intestinal mucus, increased in the supernatant of *P. freudenreichii* culture (SPFC)-treated LS 174 cells. The SPFC and live *P. freudenreichii* (LPF) reduced the disease activity index (DAI) in the rats with DSS-induced colitis. After treatment with SPFC or LPF, the mRNA levels of typical pro-inflammatory cytokines decreased and the inflammatory state was histologically improved in the rats with DSS-induced colitis. The SPFC and LPF treatments increased the gene and protein expression levels of MUC2 in the rats with DSS-induced colitis compared with the expression levels in the negative control rats, and immunohistochemistry (IHC) showed an increase of the intestinal MUC2 level. In addition, SPFC and LPF augmented the level of propionate in the faeces of the rats with DSS-induced colitis. In conclusion, *P. freudenreichii* might improve acute colitis by restoring goblet cell number and stimulating the expression of MUC2 in intestinal goblet cells.

## Introduction

As a defence reaction, inflammation is necessary to protect the body from damage. Although inflammation removes detrimental materials to enable the body to recover^1^, it is important to avoid insufficient or excessive inflammation via regulation by a well-established immune system. Inflammatory bowel disease (IBD) arises from an uncontrolled mucosal immune system^2–5^. Intestinal homeostasis is regulated by complex factors such as immune cells, epithelial cells, and the microbiota in the intestine, and together these factors affect the integrity of the intestinal mucus layer^6^. Homeostatic imbalance in the intestine causes IBD, and the incidence of IBD is gradually increasing worldwide^4^. Intestinal disorders resulting from homeostatic imbalance mainly arise as a result of three factors^7^: first, environmental factors such as smoking, hygiene, and industrialization; second, genetic factors, which include many unknown factors, genes, and complex networks^8^; and third, dysbiosis in the colon^9^. Any harmful factors in the intestine, such as clindamycin and erythromycin, reduce the diversity of the gut microbiota, which leads to a reduction in specific commensal bacteria, such as *Firmicutes*, and to increase Bacteroidetes^10^. Subsequently, the intestinal epithelium becomes damaged, microbial infiltration increases, the level of inflammation increases, and the synthesis and secretion of mucin is reduced^11^.

Mucins are heavily glycosylated *O*-glycoproteins that are produced by secretory epithelial cells, and they make up the mucus layer that functions as a physical barrier against pathogens, harmful agents, and colorectal diseases in the gut^12^. Mucins are also critical in the prevention of IBD^13^. Among at least 20 human mucins, MUC2 is the main component of mucin in colon^14,15^. MUC2 is the only gel-forming mucin expressed in the intestine at a physiologically relevant level; however, there are other gel forming mucins in other organs such as MUC5AC, MUC5B, and MUC6^14^. In addition, genetically modified mice (MUC2−/−) developed colitis and colorectal tumours^16^. IBD is characterized by a reduction in the synthesis and secretion of MUC2^17^; thus, recovering the level of MUC2 and the formation of a firm mucus layer could improve IBD.

Many aspects of the relationship between the microbiota and human health have been studied, for instance, the differences in the gut microbiomes of obese and lean mice^18^ and the brain-gut-axis^19,20^. Early life stress induced by maternal separation causes various changes in the gut microbiota, which induces the development of diseases such as irritable bowel syndrome (IBS) in the gut^21^. IBS leads to imbalanced enteric microflora and an excessive stress response^22^. The host immune system is also affected by the gut microbiota. Commensal bacterial DNA increases the resistance of the host to specific parasites, whereas reductions in the gut microflora due to antibiotic treatment increase the susceptibility to infections by parasites^23^. However, studies on the relationship between the gut microbiota and human health are insufficient; thus, little information is available to guide the utilization of beneficial microorganisms for an alternative therapy for IBD.

Short-chain fatty acids (SCFAs), mainly acetate, propionate, and butyrate, stimulate LS 174T goblet cells to secrete MUC2^24^. Among the SCFAs, propionate is the most effective compound in stimulating mucin production. *Propionibacterium freudenreichii*, a probiotic candidate, is known for producing propionate as a by-product of lactate fermentation. *P. freudenreichii* is a bacterium with a generally recognized as safe (GRAS) status. In this study, we hypothesized that the administration of *P. freudenreichii*, which produces propionate as a major metabolite, might restore intestinal goblet cell, thereby contributing to the increase of the intestinal MUC2 expression and improving DSS-induce acute colitis. To investigate the protective effect of oral administration of live *P. freudenreichii* (LPF) against intestinal mucus layer damage in rats with DSS-induced acute colitis, we first evaluated the cytotoxicity of the supernatant of *P. freudenreichii* culture (SPFC) and its stimulatory effect on the synthesis and secretion of MUC2 in human LS 174T goblet cells *in vitro*. Subsequently, SPFC and LPF were orally administered to a rat model of DSS-induced acute colitis, and the expression level of MUC2, the prevention of DSS-induced intestinal damage, and the reduction in inflammation were investigated *in vivo*.

## Results

### Cytotoxicity of SPFC in LS 174T goblet cells

**Figure 1:**
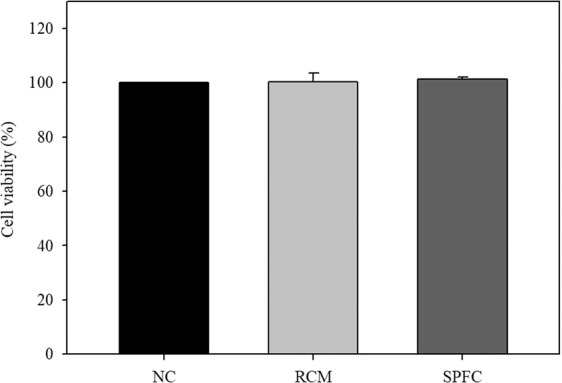
Cytotoxic effect of SPFC on LS 174T goblet cells. The cytotoxicity of SPFC was evaluated by MTT assay. The cells were treated with RCM or SPFC (10%, v/v) and incubated for 48 h. Data are expressed as the mean ± SD of three independent experiments performed in triplicate. NC: the negative control group; RCM: the reinforced clostridial medium-treated group; SPFC: the supernatant of *P. freudenreichii* culture-treated group.

To determine the cytotoxic effect of SPFC on LS 174T cells, an MTT assay was performed. RCM-cultured *P. freudenreichii* was used as the experimental control. The relative viability of the cells treated with RCM and SPFC (10%, v/v) were 100.2 ± 3.2% and 101.3 ± 0.8%, respectively, compared with the viability of the NC (100%) (Fig. 1). Therefore, SPFC, at a 10% concentration (v/v), had no cytotoxic effect on the cells.

### Effect of SPFC on the gene and protein expression levels of MUC2 in LS 174T cells

**Figure 2:**
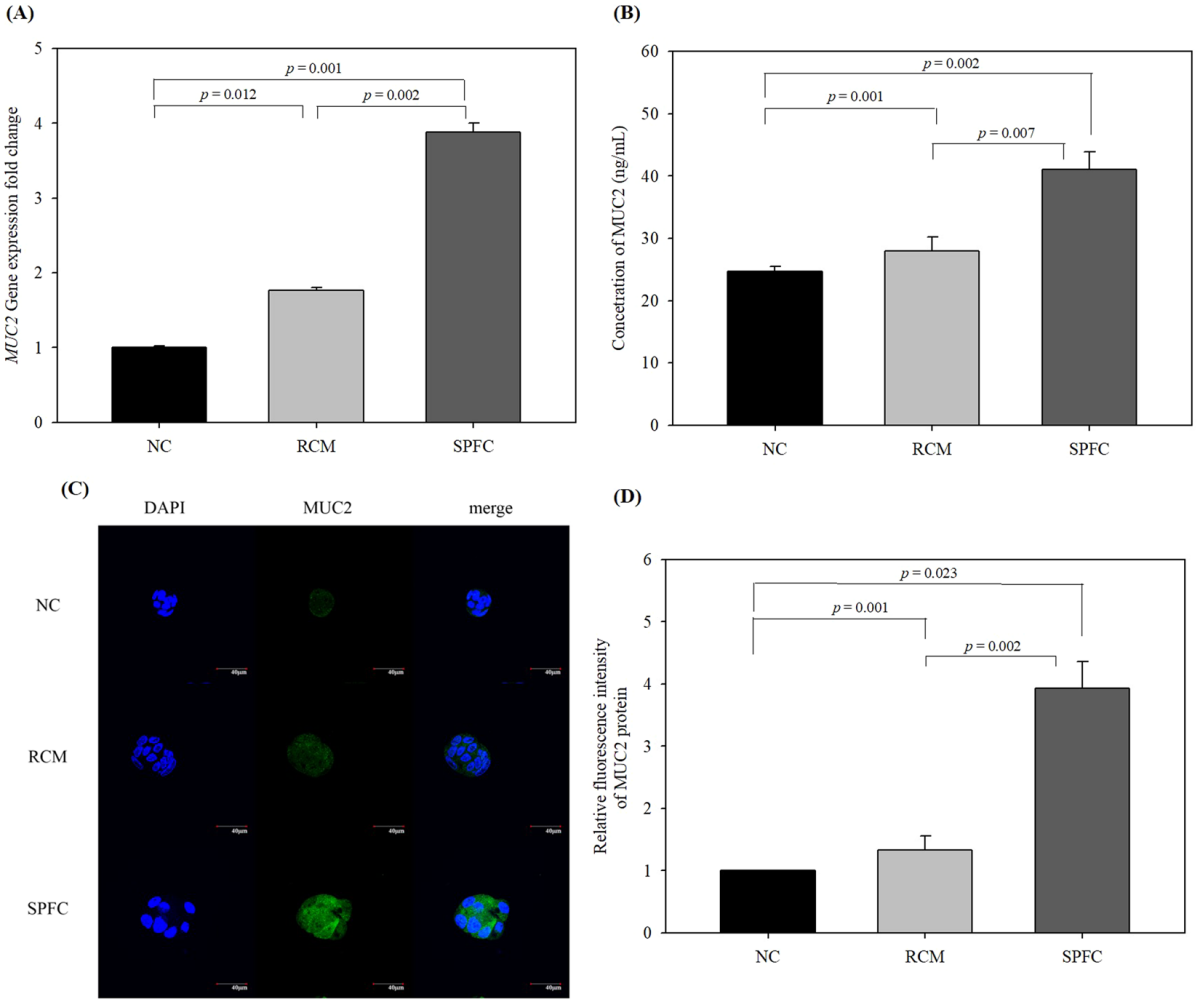
mRNA and protein expression levels of MUC2 in SPFC-treated LS 174T cells. The cells were treated with RCM or SPFC (10%, v/v) and incubated for 48 h. (**A**) mRNA expression level determined by qPCR; (**B**) MUC2 concentration in the supernatant of the cell culture determined by ELISA; (**C**) images of immunofluorescence-stained cells; (**D**) quantification results of MUC2 production by immunocytochemistry. mRNA expression levels were normalized to the reference gene *GAPDH*. Each value indicates the mean ± SD of three independent experiments performed in triplicate. The significance of the differences was determined by the Student’s *t*-test. NC: the negative control group; RCM: the reinforced clostridial medium-treated group; SPFC: the supernatant of *P. freudenreichii* culture-treated group.

The stimulatory effect of SPFC on MUC2 expression in LS 174T cells was measured by qPCR (mRNA level) and by enzyme-linked immunosorbent assay (ELISA) and immunocytochemistry (protein level). The expression level of the *MUC2* gene was normalized to the expression level of *glyceraldehyde-3-phosphate dehydrogenase* (*GAPDH*). Unexpectedly, the RCM-treated LS 174T cells exhibited an increased expression level of *MUC2* compared with the expression level in the NC. The expression level of *MUC2* in the SPFC-treated cells significantly increased by 2.20- and 3.88-fold compared with the expression level in the RCM-treated cells and the NC, respectively (Fig. 2A). The ELISA results of the protein expression level revealed that the secreted MUC2 protein in SPFC-treated cells significantly increased by 1.46- and 1.66-fold compared with the expression in RCM-treated cells and the NC, respectively (Fig. 2B). In addition, we measured the effect of SPFC on the expression of MUC2 protein in LS 174T cells by an immunofluorescence-staining assay (Fig. 2C). Fluorescence intensity was quantified and normalized to the NC (set as 1) (Fig. 2D). As shown in the gene expression, the cells treated with RCM showed an increase in fluorescence intensity compared with the fluorescence intensity of the NC. However, the fluorescence intensity of the SPFC-treated cells increased significantly by 2.95- and 3.93-fold, compared with the RCM-treated cells and the NC, respectively. The results suggested that SPFC significantly stimulated MUC2 expression at the transcriptional and translational levels.

### LPF and SPFC protect the epithelial layer and conserve goblet cells in the crypts of the colon in rats with DSS-induced acute colitis

**Figure 3:**
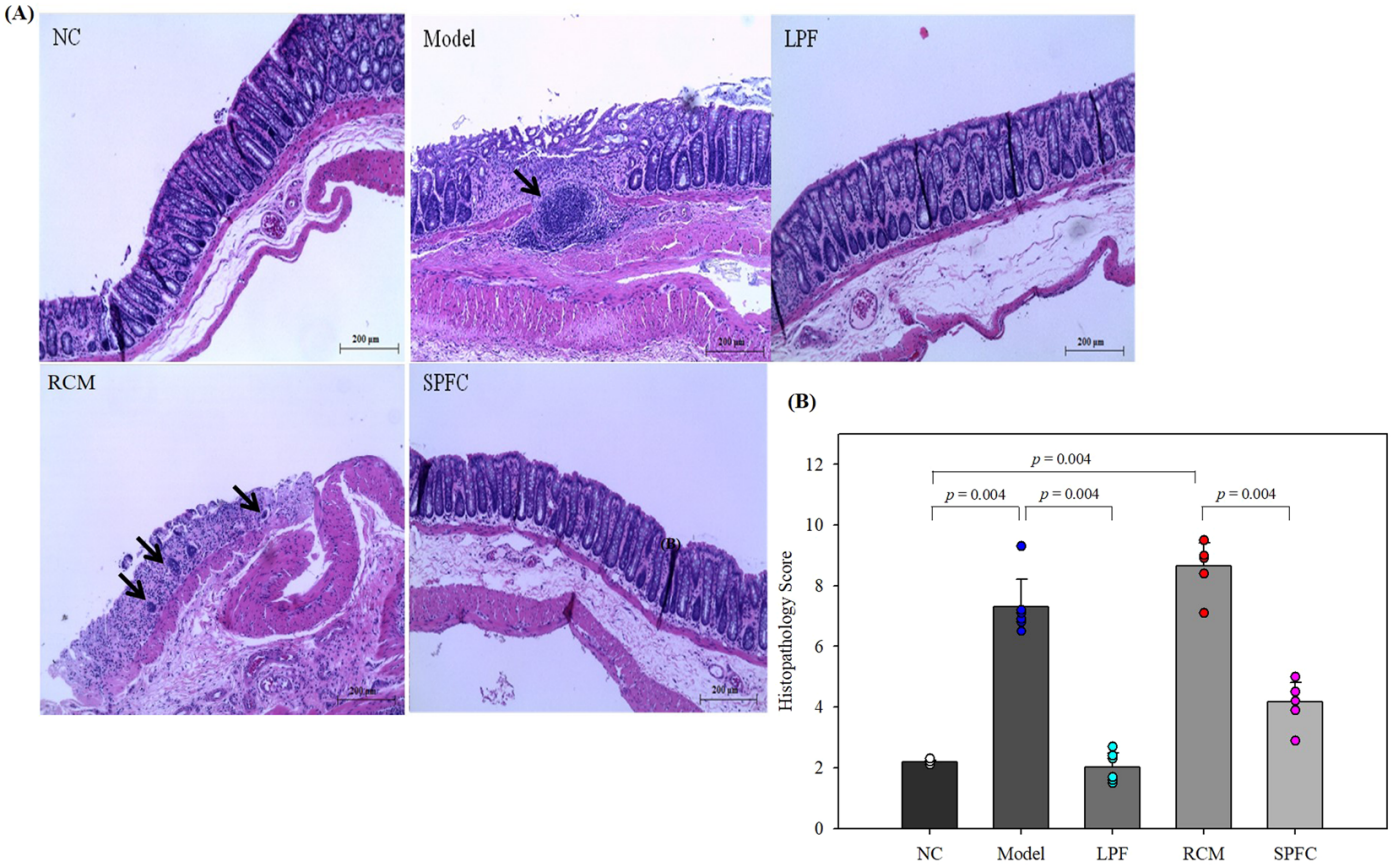
Effects of LPF and SPFC on the alleviation of inflammation in rats with DSS-induced acute colitis. The middle portions of distal colons were stained with H&E (**A**) and the histopathology score was assessed (**B**) in rats with DSS-induced acute colitis (n = 6 per group, at least 5 parts were evaluated for each sample). The scores are expressed as the mean ± SD. Mann-Whitney test was performed. The black arrows indicate infiltrated cells. NC: the negative control group; Model: the 5% DSS-induced colitis group; LPF: the 5% DSS and live *P. freudenreichii*-treated group; RCM: the 5% DSS and reinforced clostridial medium-treated group; SPFC: the 5% DSS and supernatant of *P. freudenreichii* culture-treated group.

To investigate the protective effect of LPF and SPFC on the destruction of the crypt structure, which could result in a decrease in the number of intact goblet cells, LPF and SPFC were orally administered to rats with acute colitis induced by 5% DSS (w/v) (Supplementary Fig. S1). Middle portions of the distal colon of each rat were dissected and stained with haematoxylin and eosin (H&E) and alcian blue (AB), and the loss of surface epithelium, the destruction of crypt structures, and the infiltration of inflammatory cells were estimated according to Supplementary Table S1. The model group showed a seriously damaged surface epithelium, distorted crypt structure, and prevalently infiltrating inflammatory cells (Fig. 3A); thus, the group’s histopathology score was 7.30 ± 0.92 (Fig. 3B). In addition, the distal colons of the model group showed a partial abscess or ulceration. The RCM group showed a similar degree of damage as the model group (histopathology score: 8.65 ± 0.76). However, the LPF group (histopathology score: 2.03 ± 0.45) showed similar histological characteristics as those of the NC group (histopathology score: 2.18 ± 0.07). The distal colons of the rats in the SPFC group showed minor destruction of the crypt structures and increased infiltration of inflammatory cells to a small extent (histopathology score: 4.17 ± 0.66).

**Figure 4:**
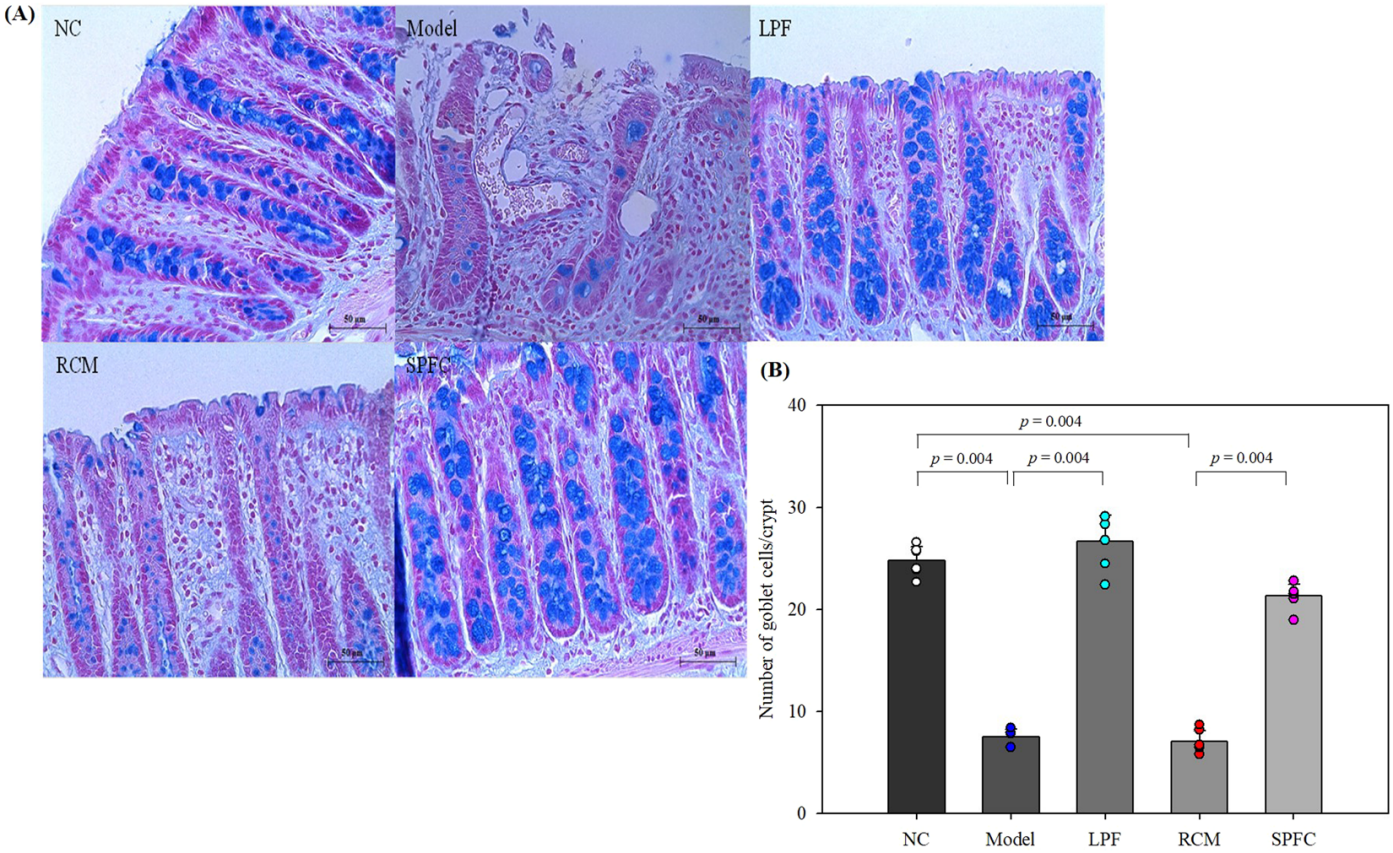
Protective effects of LPF and SPFC against goblet cell destruction in rats with DSS-induced acute colitis. The middle portions of distal colons were stained with alcian blue (**A**), and the numbers of goblet cells in the crypts were counted (**B**) in rats with DSS-induced acute colitis. (n = 6 per group, at least 5 parts were evaluated for each sample). The numbers of goblet cells/crypts are expressed as the mean ± SD. Mann-Whitney test was performed. NC: the negative control group; Model: the 5% DSS-induced colitis group; LPF: the 5% DSS and live *P. freudenreichii*-treated group; RCM: the 5% DSS and reinforced clostridial medium-treated group; SPFC: the 5% DSS and supernatant of *P. freudenreichii* culture-treated group.

The number of intact goblet cells per crypt in the rats with DSS-induced acute colitis was counted by AB staining. The model and RCM groups exhibited a significantly decreased number of goblet cells (7.50 ± 0.73 and 7.08 ± 1.02, respectively) compared with the number of goblet cells in the NC group (24.78 ± 1.35) (Fig. 4). On the other hand, the numbers of goblet cells in the LPF and SPFC groups were 26.72 ± 2.51 and 21.32 ± 1.16, respectively, which were similar to the number in the NC group. The results suggested that the LPF and SPFC treatments prevented the destruction of the epithelial layer of the colon in the rats with DSS-induced acute colitis.

### LPF and SPFC stimulate the expression of MUC2 in rats with DSS-induced acute colitis

**Figure 5:**
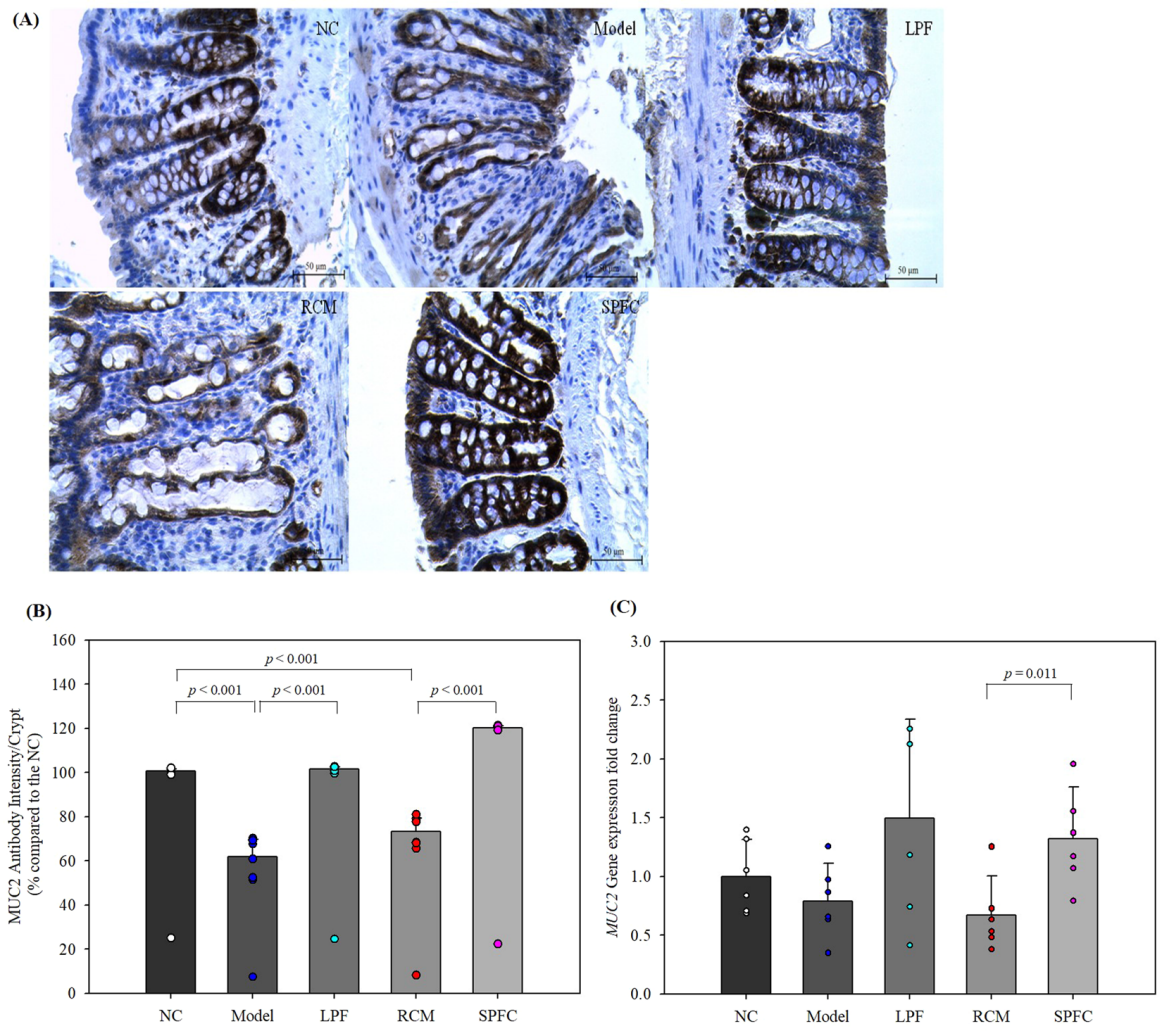
LPF- and SPFC-induced increases in the expression level of MUC2 in the colon in rats with DSS-induced acute colitis. The middle portions of distal colons were analysed for the expression of MUC2 by IHC (**A**), and the intensity of MUC2 expression was quantified (**B**) (n = 6 per group, at least 5 parts were evaluated for each sample). The expression levels of the *MUC2* gene were measured by qPCR (**C**). The data are expressed as the mean ± SD. Mann-Whitney test was performed. NC: the negative control group; Model: the 5% DSS-induced colitis group; LPF: the 5% DSS and live *P. freudenreichii*-treated group; RCM: the 5% DSS and reinforced clostridial medium-treated group; SPFC: the 5% DSS and supernatant of *P. freudenreichii* culture-treated group.

After observing the conservation of the goblet cells in the LPF and SPFC groups, the expression level of MUC2 was measured to confirm the protective effect of the LPF and SPFC against acute colitis. The gene and protein expression levels of MUC2 were measured by qPCR and IHC, respectively. The IHC results revealed that the protein expression of MUC2 in the model and RCM groups decreased to 61.93 ± 8.91% and 73.32 ± 7.04%, respectively, compared with the expression in the NC group (100 ± 1.27%) (Fig. 5A,B). The MUC2 expression of the LPF group (101.48 ± 1.32%) was similar to that of the NC group, and the MUC2 expression in the SPFC group increased significantly to 120.34 ± 0.96% compared with the expression in the NC group. MUC2 expression increased 1.64-fold in both the LPF and SPFC groups compared with the expression in the model and RCM groups. *MUC2* gene expression was reduced by 0.79- and 0.67-fold in the model and RCM groups compared with the expression in the NC group, whereas the expression in the LPF and SPFC groups increased by 1.50- and 1.32-fold, respectively, compared with the expression in the NC group (Fig. 5C). *MUC2* expression increased 1.90- and 1.97-fold in the LPF and SPFC groups, respectively, compared with the expression in the model and RCM groups. The results were consistent with the conservation of goblet cells in the colons of the rats with DSS-induced acute colitis.

### LPF and SPFC alleviate DSS-induced acute colitis in rats

**Figure 6:**
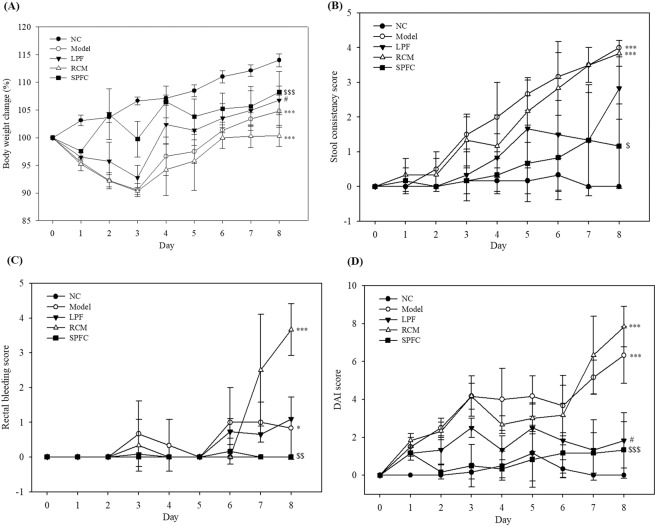
Disease-relieving effects of LPF and SPFC in rats with DSS-induced colitis. Daily changes in body weight (**A**), stool consistency (**B**), and rectal bleeding (**C**) of all of the rats were observed and evaluated during the DSS treatment period. Body weights were calculated by dividing the body weight on each day by the initial body weight on the first day before DSS treatment and are expressed as a group percentage mean ± SD (n = 6 per group). The disease activity index (DAI) was scored by summing scores of the three parameters (**D**). The values are expressed as the mean ± SD (n = 6 per group). The significance of body weight, stool consistency, rectal bleeding and DAI on day 8 are marked. Mann-Whitney test was performed. *p < 0.05, ***p < 0.001, compared with the NC group; ^#^*p* < 0.05, compared with the model group; ^$^*p* < 0.05, ^$$^*p* < 0.01, and ^$$$^*p* < 0.001 compared with the RCM group. NC: the negative control group; Model: the 5% DSS-induced colitis group; LPF: the 5% DSS and live *P. freudenreichii*-treated group; RCM: the 5% DSS and reinforced clostridial medium-treated group; SPFC: the 5% DSS and supernatant of *P. freudenreichii* culture-treated group.

To evaluate the DAI, the body weight, stool consistency, and rectal bleeding were observed daily after DSS supplementation in drinking water, and the experiment was terminated on the 8^th^ day after DSS supplementation because the group treated with DSS only (the model group) showed severe diarrhoea and bleeding. All groups except the NC group exhibited decreased body weight 3 days after treatment with DSS; however, the body weight in all the groups gradually increased after 3 days (Fig. 6A). The rats with DSS-induced colitis that were treated with LPF or SPFC showed more body weight gain than the rats in the model and RCM groups. The stool condition and bleeding status were observed daily and scored according to Supplementary Table S2. The faecal pellets of the model group started to become watery on day 3, and some of the rats had severe diarrhoea on day 6 after DSS supplementation (Fig. 6B). The rats with DSS-induced colitis that were treated with LPF or SPFC had less diarrhoea than the rats in the model and RCM groups. The rats in the model and RCM groups presented with blood in the faeces on day 3 after DSS supplementation (Fig. 6C). These symptoms became aggressively worse in the model and RCM groups, whereas the LPF and SPFC groups did not show any severe symptoms. Based on the results of body weight change, stool consistency, and rectal bleeding, the DAI scores of the NC, model, RCM, LPF, and SPFC groups were 0, 6.33, 7.83, 2.50, and 1.33, respectively on day 8 after DSS supplementation (Fig. 6D). The results suggested that LPF and SPFC might improve DSS-induced acute colitis in rats.

### Protective effects of LPF and SPFC against decreased colon length in rats with DSS-induced acute colitis

The colon lengths of the rats in the model and RCM groups, 10.47 ± 1.53 cm and 12.25 ± 0.76 cm, respectively, were significantly shorter than those of the rats with the NC group (14.78 ± 0.97 cm). However, the colon lengths of the rats in the LPF and SPFC groups recovered to 15.12 ± 1.66 cm and 15.97 ± 0.31 cm, respectively (Supplementary Fig. S2A,B). These results suggested that the LPF and SPFC restored colon length to the normal range. The results suggested that LPF and SPFC treatment decreased the colon damage in the rats with DSS-induced acute colitis.

### LPF and SPFC reduce expression levels of pro-inflammatory cytokines and SPFC increases expression level of an anti-inflammatory cytokine in rats with DSS-induced acute colitis

**Figure 7:**
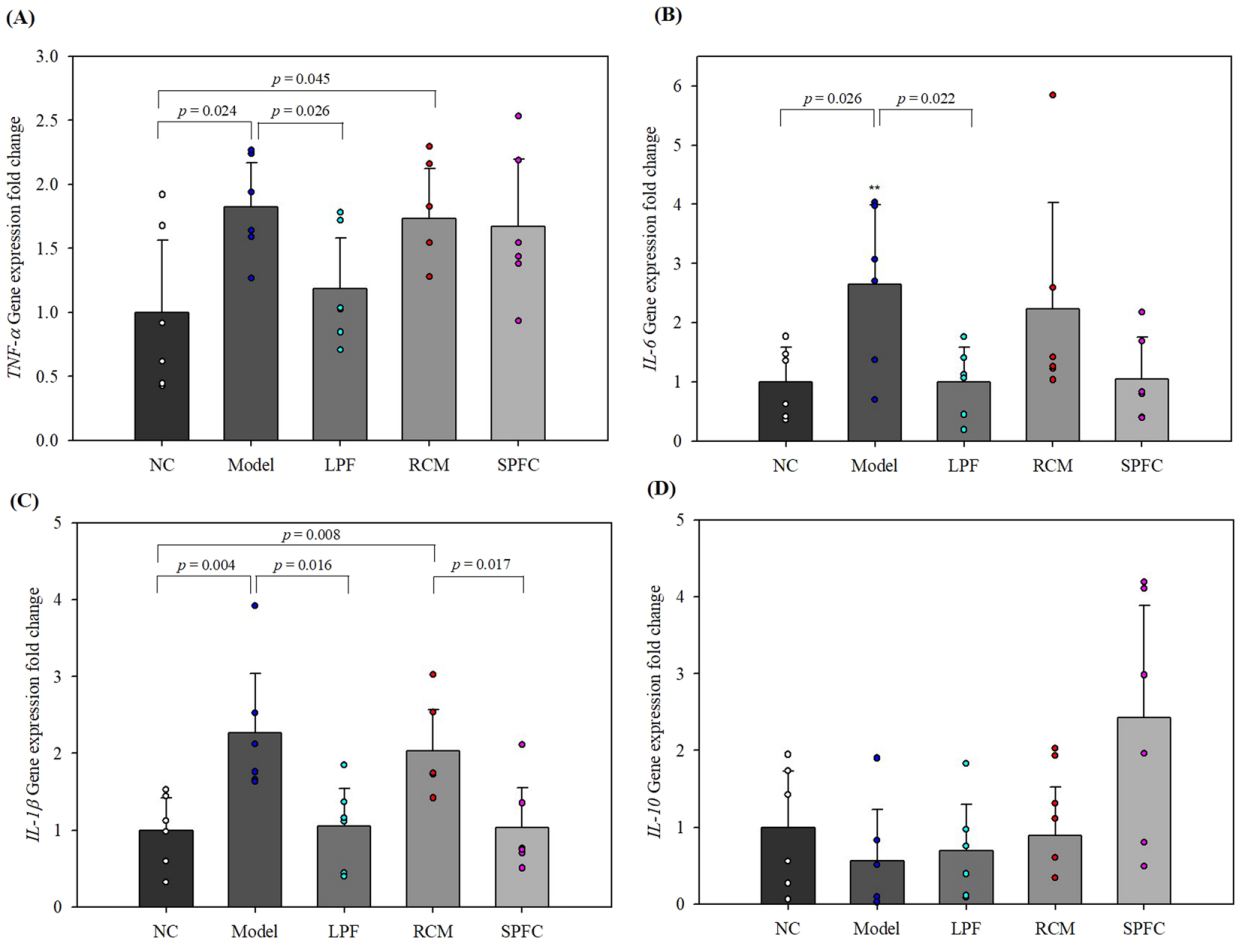
LPF- and SPFC-induced decreases in pro-inflammatory cytokines and increases an anti-inflammatory cytokine in the distal colon of rats with DSS-induced colitis. The expression levels of pro-inflammatory cytokines (**A**) *TNF-α*, (**B**) *IL-6*, (**C**) *IL-1β*, and anti-inflammatory cytokine (**D**) *IL-10* were measured by qPCR. Data are expressed as the mean ± SD (n = 6 per group). NC: the negative control group; Model: the 5% DSS-induced colitis group; LPF: the 5% DSS and live *P. freudenreichii*-treated group; RCM: the 5% DSS and reinforced clostridial medium-treated group; SPFC: the 5% DSS and supernatant of *P. freudenreichii* culture-treated group.

As inflammation in the rats with DSS-induced acute colitis was observed by H&E staining, to evaluate the inhibitory effect of the LPF and SPFC on the expression of the typical pro-inflammatory cytokines, TNF-α, IL-6, and IL-1β and the stimulating effect of the LPF and SPFC on the expression of IL-10, an anti-inflammatory cytokine, the mRNA expression levels of the cytokines in the distal colons of the rats in each group were measured by quantitative real-time polymerase chain reaction (qPCR). The expression levels of *TNF-α*, *IL-6*, and *IL-1β* in the model group were significantly elevated by 1.82-, 2.64-, and 2.27-fold, respectively, compared with the expression levels in the NC group, while the expression levels of *TNF-α*, *IL-6*, and *IL-1β* in the LPF group were 1.19-, 1.00-, and 1.06-fold, respectively, compared with the expression levels in the NC group (Fig. 7A–C). The expression levels of *TNF-α*, *IL-6*, and *IL-1β* in the LPF group decreased by 35.0%, 62.1%, and 53.5%, respectively, compared with the expression levels in the model group. The expression levels of *TNF-α*, *IL-6*, and *IL-1β* in the RCM group were significantly increased by 1.73-, 2.23-, and 2.03-fold, respectively, compared with the expression levels in the NC group, whereas the expression levels of *TNF-α*, *IL-6*, and *IL-1β* in the SPFC group (1.67-, 1.05-, and 1.03-fold, respectively) were lower than those in the RCM group. The expression levels of *TNF-α*, *IL-6*, and *IL-1β* in the SPFC group decreased by 3.5%, 52.9%, and 49.3%, respectively, compared with the expression levels in the RCM group. The results suggested that LPF and SPFC had anti-inflammatory activities. Although the expression level of *IL-10* was not significant, the expression level increased 1.22-fold in the LPF compared with the model group and increased 2.73-fold in the SPFC group compared with the RCM group (Fig. 7D).

### Changes in SCFAs in the faeces of rats with DSS-induced acute colitis after treatment with LPF or SPFC

**Figure 8:**
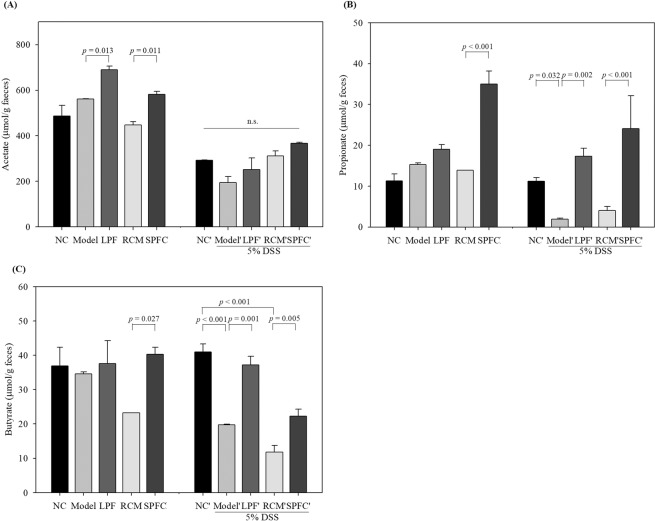
SCFA concentrations increased in the colons of the LPF- and SPFC-treated rats with DSS-induced acute colitis. SCFA concentrations in the rat faeces were measured by HPLC, and the data are expressed as the mean ± SD (μmol/g faeces). Acetate (**A**), propionate (**B**), and butyrate (**C**) concentrations before (left) and after (right) treatment with DSS. NC: the negative control group; Model: the 5% DSS-induced colitis group; LPF: the 5% DSS and live *P. freudenreichii*-treated group; RCM: the 5% DSS and reinforced clostridial medium-treated group; SPFC: the 5% DSS and supernatant of *P. freudenreichii* culture-treated group.

*P. freudenreichii* is a propionate-producing bacterium; thus, to investigate the effects of the LPF and SPFC on SCFA production in the colons of the experimental rats, HPLC was performed with the faeces of the rats with DSS-induced acute colitis. The concentration of acetate before the rats were provided with DSS was higher in the LPF and SPFC groups (690.52 ± 15.61 and 581.26 ± 13.50 µmol/g faeces, respectively) than in the NC group (486.45 ± 47.63 µmol/g faeces), the model group (561.30 ± 1.72 µmol/g faeces), and the RCM group (447.54 ± 14.79 µmol/g faeces) (Fig. 8A). Although the concentration of acetate increased in the LPF and SPFC groups, only the SPFC group showed a significant increase compared with the concentration in the RCM group. After DSS administration, the concentration of acetate in all groups decreased, and there was no significant difference in the concentration of acetate between the groups. The concentrations of propionate in the LPF and SPFC groups increased significantly to 19.01 ± 1.14 µmol/g faeces and 34.99 ± 3.18 µmol/g faeces, respectively, compared with the concentration in the NC group (11.32 ± 1.66 µmol/g faeces) before DSS administration (Fig. 8B). After the rats were provided with DSS, the concentrations of propionate in the rats in all groups except the NC group were lower than those in the rats before DSS administration. The concentrations of propionate in the model and RCM groups were 1.88 ± 0.32 µmol/g faeces and 4.04 ± 0.94 µmol/g faeces, respectively, whereas the LPF (17.31 ± 1.93 µmol/g faeces) and SPFC (24.07 ± 8.05 µmol/g faeces) groups showed higher concentrations of propionate than the model and RCM groups, respectively. Before the rats were provided with DSS, the concentrations of butyrate in the faeces in the NC, model, LPF, RCM, and SPFC groups were 36.89 ± 5.43 µmol/g faeces, 34.62 ± 0.52 µmol/g faeces, 37.55 ± 6.76 µmol/g faeces, 23.24 ± 0.02 µmol/g faeces, and 40.29 ± 2.00 µmol/g faeces, respectively (Fig. 8C), which revealed a similar concentration of butyrate in all the groups except the RCM group. After DSS treatment, the concentrations of butyrate in the model (19.71 ± 0.27 µmol/g faeces) and RCM (11.77 ± 1.92 µmol/g faeces) groups decreased significantly compared with the concentrations in the NC group (40.94 ± 2.37 µmol/g faeces), while the LPF (37.21 ± 2.53 µmol/g faeces) and SPFC (22.27 ± 2.06 µmol/g faeces) groups showed significantly higher concentrations than the model and RCM groups, respectively. Acetate decreased in the NC and LPF groups after treatment with DSS. Unlike acetate, the propionate and butyrate levels in the model and RCM groups decreased significantly in the rats with DSS-induced acute colitis compared with the levels in the rats in the NC group, while the propionate and butyrate levels in the LPF and SPFC groups were significantly higher than those in the model and RCM groups, respectively. SPFC contained propionate, which may affect the raise faecal propionate concentration in rats treated with SPFC.

### Amount of *Propionibacterium* in the gut after the administration of LPF

To confirm that the orally provided LPF reached and settled in the gut, the colonies of *Propionibacterium* in the faeces of the LPF group were counted using yeast extract sodium lactate agar (YELA) selective medium. The samples from days 7, 14, 21, and 29 were used. The amount of *Propionibacterium* on day 7, the last day of the adaptation period, was below the detectable range (Supplementary Fig. S3). However, after 10^8^ CFU of LPF was orally administered for 7 days (day 14), the amount of *Propionibacterium* rapidly increased to 1.24 × 10^5^ CFU/g faeces. The bacterium was maintained at almost a constant level until day 21 (1.16 × 10^5^ CFU/g faeces). On the last day of 5% DSS treatment to induce colitis, day 29, the amount of *Propionibacterium* increased slightly to 3.80 × 10^5^ CFU/g faeces. Although there was some growth of other bacteria on the YELA medium, the results suggested that orally administered LPF reached the gut and became established.

## Discussion

Currently, considering the interest in the interaction between the host and the gut microbiota, research showing the beneficial effects of probiotics for human health are widespread. However, the mechanism of the beneficial bacteria-induced improvements in the various diseases is still unclear. The probiotics *Lactobacillus plantarum* and *Lactobacillus rhamnosus* induce an increase in mucin gene expression in HT-29 human intestinal epithelial cells, resulting in the inhibition of adherence of enteropathogenic *Escherichia coli* to intestinal epithelial cells^25^. In contrast, colon epithelial HT-29 cells do not produce mucin *in vitro*^26^. Cheeses made by a starter containing *P. freudenreichii* ITG P20 (CIRM-BIA 129) decrease the severity of trinitrobenzenesulfonic acid (TNBS)-induced acute colitis through anti-inflammatory activity and by reducing colonic oxidative stress and epithelial cell damage^27^. In this study, a mucin-producing goblet cell line was used, and *P. freudenreichii* increased MUC2 expression *in vitro* and *in vivo*. In addition, a GRAS bacterium, *P. freudenreichii*, increased MUC2 expression at the transcriptional and translational levels *in vivo* and restored the intestinal goblet cell number, resulting in improvements in DSS-induced acute colitis. Besides *P. freudenreichii*, some dairy propionate producers show beneficial effects on human health. *Lactobacillus mucosae* can adhere to intestinal mucus and inhibits pathogen’s growth in the gastrointestinal tract, thereby considering as a potential probiotic candidate^28^. *Phascolarctobacterium faecium* abundantly colonized in the human gastrointestinal tract is associated with the positive mood of the human^29^. On the other hand, increased abundance of some propionate-producing bacteria such as *Veillonella parvula*, *Bacteroides eggerthii*, *Bacteroides fragilis*, *Ruminococcus bromii*, and *Eubacterium dolichum* may be related to relapsing polychondritis, an inflammatory disease in cartilaginous tissues of the whole body, by stimulation of intestinal Th17 cells and induction of Th17/Treg cell imbalance in the intestine^30^.

Disruption of the intestinal mucus layer induces colitis, which is accompanied by inflammation; thus, restoring the mucus layer improves colitis and usually the accompanying inflammation. In this study, the LPF and SPFC restored the intestinal goblet cell number accompanying with the increase of MUC2 production that was damaged by DSS and reduced the gene expression of inflammatory cytokines, which indicates that the LPF and SPFC improved colitis. The DAI showed consistent results; the LPF and SPFC improved colitis in the rats with DSS-induce acute colitis. Consequently, *P. freudenreichii* protects the host gut environment from DSS-induced acute colitis and also produces an anti-inflammatory effect. The intestinal gland is covered by an epithelial layer that contains various types of cells, including goblet cells, that produce mucin. Goblet cells are located in colonic crypts, which are the structure of the intestinal gland in colon. IBD leads to progressive intestinal crypt destruction, which induces a reduction in goblet cells and the destruction of the intestinal mucus layer. In this study, the LPF and SPFC contributed to conserve colonic crypt structure, which allowed goblet cells to express and secrete MUC2 protein.

In many studies of SCFAs, butyrate is chiefly spotlighted due to its variety of roles and activities such as acting as an energy source, maintaining intestinal homeostasis, and inhibiting histone deacetylase activity^31,32^. Propionate is a well-known SCFA that improves intestinal inflammation^33^, and propionate produced by gut microbiota contributes to various human health, which goes beyond the gut. Propionate has been shown to function in inhibiting the biosynthesis of hepatic cholesterol, regulating food intake and obesity, and inducing hypophagia, which are related to metabolism and energy homeostasis^34,35^. Propionate also lowers lipogenesis and carcinogenesis risk, protects from hypertensive cardiovascular damage, and improves multiple sclerosis^36–38^. However, it is likely that the health-promoting effects of propionate are less studied than those of butyrate. On the other hand, propionate shows hepatotoxicity at the concentration over 70 μM^39^. Therefore, the administration of a high concentration of propionate might not be an effective method to obtain beneficial effects. The administration of LPF is an alternative method to ensure a steady supply of propionate in the gut. *P. freudenreichii* is mainly used as a starter bacterium in the processing and production of cheese and produces acetate and propionate as by-products. In this study, the LPF and SPFC groups showed higher propionate levels than the NC group. However, the concentrations of propionate in the gut in the LPF and SPFC groups were lower than the concentrations that induce hepatotoxicity.

Prebiotic fibres show many beneficial effects including amelioration of inflammation in the colon and SCFAs produced from the fibres by SCFA-producing gut microbiota contribute to the beneficial effects. Although SCFAs, especially butyrate, have been known the beneficial effect on colitis, a recent study showed that the negative effect of butyrate on colitis in animal study^40^. Fermentable fibres altered microbiota composition, which induced excess butyrate production resulted in exacerbation of IBD. Thus, the positive role of SCFA in colitis seems to fluctuate depending on alterations in the gut microbiome. In this study, the concentrations of butyrate in LPF- and SPFC-treated rats did not exceed the concentration of the negative control. In addition, although the concentration of propionate significantly increased compared with the negative control, we did not observe the amelioration of colitis but improvement of colitis in this study. However, we need to analyze the change of gut microbiota composition by LPF and SPFC treatment in the further study.

In this study, certain RCM components might be carried over to SPFC; thus, RCM was used for the experimental control. Although it was not statistically significant, RCM showed a weak ability to augment the MUC2 expression level *in vitro*. RCM contains acetate (3 g/L), and acetate is also known to slightly increase MUC2 expression in LS 174T cells; this action may have induced a slight increase in the expression level of MUC2. Although the DAI scores of the LPF and SPFC groups were not significantly different, other results were different between them in terms of histopathology score and cytokine expression, especially, the lack of inhibitory effect of SPFC on *TNF-α* expression. A common *Lactobacillus* culture medium, de Man Rogosa Sharpe (MRS), affects cytokine (for instance, IL-10 and IL-12p70) production^41^. Roswell Park Memorial Institute (RPMI) medium also has effects on cytokine (IL-10 and IL-12) production^42^. Therefore, the RCM itself might have exerted some effects on cytokine production, which may have resulted in a difference in TNF*-α* expression. Thus, the RCM may have interfered with the effect of the bacterial metabolites in the SPFC group. Further study is needed to explain the different inhibitory effects of LPF and SPFC on the expression of *TNF-α*. Although it was not significant, the expression level of an anti-inflammatory cytokine, IL-10, increased in the SPFC group, but not in the LPF group, which suggests that *P. freudenreichii* might be more effective in inhibiting the expression of pro-inflammatory cytokines than in stimulating the expression of anti-inflammatory cytokines.

SlpB, a surface protein of *P. freudenreichii*, plays a role in the adhesion of the bacterium to the human colon adenocarcinoma cell line HT-29^26^. The oral administration of LPF increased the amount of *Propionibacterium* in the intestines of the LPF group, which means that LPF is expected to have been established and to produce propionate in the gut, which resulted in an increase in the propionate level in the LPF group compared with that in the NC group. In this study, specific pathogen-free (SPF) rats were used, which means that although there is some fluctuation in the composition of the microbiota, the microbiota in their gut has already been established. Although the colonies grown on *Propionibacterium*-selective medium increased in the intestines of the LPF group, it is not clear that only *P. freudenreichii* was grown on the medium. Therefore, to verify that *P. freudenreichii* was established in the gut and that it induces an improvement in acute colitis, further studies are needed using germ-free animals. In addition, although the LPF and SPFC showed little difference in inducing improvements in DSS-induced acute colitis, their administration improved acute colitis. Therefore, the effective factor(s) in LPF and SPFC should be identified in the further studies.

Butyrate and propionate increase *MUC2* mRNA expression in LS 174T cells through the acetylation/methylation of the histone at the MUC2 promoter^35^. *P. freudenreichii* kills cells from the human colorectal carcinoma cell lines, HT-29 and Caco-2, by apoptosis, which is induced by the propionate and acetate that are produced by the bacterium as major cytotoxic agents^43^. Both LPF and SPFC exhibit similar effects in the improvement of DSS-induced colitis, which means that LPF and SPFC may or may not contain common factor(s). *P. freudenreichii* produces several beneficial metabolites including SCFAs, especially propionate. In this study, the LPF and SPFC groups showed higher propionate and butyrate levels after the induction of colitis with DSS than the model and RCM groups, respectively. The butyrate level did not decrease in the NC group after DSS administration, which may be due to many butyrate-producing bacteria in the gut^44^. Unlike the butyrate levels, the propionate levels in the LPF and SPFC groups increased significantly compared with the levels in the NC group. Therefore, the propionate in SPFC and the propionate produced by the administration of LPF may be one of the common factor(s) that contributed to the restoration of goblet cell number that resulted in the improvement of acute colitis by stimulating MUC2 expression and reducing inflammation.

In conclusion, dairy *P. freudenreichii* has probiotic characteristics including the potential to kill colon cancer cells. In addition, a novel explanation of the effect of *P. freudenreichii* on the alleviation of colitis was provided in this study. This work highlights the potential of *P. freudenreichii* as a probiotic to improve IBD in a safe and efficient way by restoring the intestinal goblet cell number accompanying with the increase of MUC2 expression as well as exerting anti-inflammatory effects in the distal gut environment.

## Materials and Methods

### Materials

RPMI-1640 medium, foetal bovine serum (FBS), and penicillin/streptomycin for the cultivation of cells were obtained from HyClone (Logan, UT, USA). Reinforced clostridial medium (RCM) was purchased from Oxoid (Hampshire, UK), 3-(4,5-dimethylthiazol-2-yl)-2,5-diphenyltetrazolium bromide (MTT) was obtained from Amresco (Solon, OH, USA), and dextran sodium sulfate (DSS, MW 36,000–50,000 Da) was purchased from MP Biomedicals (Solon, OH, USA). High-performance liquid chromatography (HPLC)-grade acetate, propionate, and butyrate; dimethyl sulfoxide (DMSO); and 4′,6-diamidino-2-phenylindole (DAPI) were obtained from Sigma (St. Louis, MO, USA).

### Cell culture

Human LS 174 T goblet cells obtained from the Korean Cell Line Bank (Seoul, Korea) were cultured in RPMI-1640 medium supplemented with 10% FBS, 100 units/mL penicillin and 100 μg/mL streptomycin at 37 °C in an atmosphere of 5% CO_2_ and 95% air, and the media was replaced with fresh media every two days.

### Preparation of LPF and SPFC

The *P. freudenreichii* KCTC 1063 strain (Korean Collection for Type Cultures, Jeongeup-si, Korea) was grown in RCM at 37 °C for 36–40 h in an anaerobic chamber with a BD GasPak™ EZ container system (Becton Dickinson, Sparks, MD, USA). After 3 activation periods, live *P. freudenreichii* (LPF) was harvested by centrifugation at 3,000 × *g* for 5 min at 4 °C, washed twice with phosphate-buffered saline (PBS), and diluted to a concentration of 10^8^ colony-forming units (CFUs)/mL with PBS. The supernatant of the *P. freudenreichii* culture (SPFC) was adjusted to a pH 7.0 ± 0.1 using a small amount of 5 M NaOH and filtered with a membrane filter (0.22 μm pore size) (Sartorius, Goettingen, Germany). The concentration of propionate in SPFC was 32.7 mM and the chromatogram is shown in Supplementary Fig. S4. For an experimental control, RCM was incubated and processed in the same manner as SPFC to exclude the possible effects of any factors derived from RCM on the LS 174T cells.

### Measurement of cytotoxicity of SPFC in LS 174T cells

Pre-confluent LS 174 T cells were seeded in 96-well plates and treated with 10% (v/v) SPFC or RCM in RPMI medium for 48 h. The medium was aspirated, and cells were incubated in MTT diluted with RPMI medium for 1 h at 37 °C. Subsequently, the formazan crystals that were produced were solubilized in 100 μL of DMSO for up to 1 h at room temperature. The absorbance was measured at 540 nm using a SpectraMax 340 microplate reader (Molecular Devices Corp., Sunnyvale, CA, USA). The relative cell viability (%) was calculated with the following equation: cell viability (%) = [OD (experiment group)/OD (control group)] × 100.

### ELISA

To measure the secreted MUC2, the supernatants of the LS 174 cell culture were prepared by centrifugation at 1,000 × *g* at 4 °C. The concentration of MUC2 protein in the supernatants was measured according to the instructions from the Human MUC2 ELISA kit obtained from Elabscience (E-EL-H0632, Houston, TX, USA).

### Immunocytochemistry

The cells were fixed with 4% paraformaldehyde and permeabilized by 0.1% Triton X-100 for 10 min. After being blocked with 10% normal donkey serum, the cells were incubated with a primary antibody against MUC2 (1:1,000, GTX100664, Genetex, Irvine, CA, USA) at 4 °C overnight. Goat anti-rabbit IgG (H + L), DyLight 488 (1:1,000, 35552, Thermo Scientific) was used as a secondary antibody, and DAPI (1:10,000) was applied to counterstain the nuclei for 10 min at room temperature. Samples were washed with PBS three times for 5 min, and the coverslips were placed onto slides and mounted using VECTASHIELD® (Vector Laboratories, Burlingame, CA, USA). Images were obtained using a Nikon C1 plus confocal laser scanning microscope (Nikon, Tokyo, Japan).

### DSS-induced acute colitis rat model

Male Sprague-Dawley rats 5 weeks of age with an average weight of 147.50 ± 5.53 g were purchased from Koatech (Pyeongtaek, Korea). The rats were housed and maintained at a constant temperature, 24 ± 1 °C, and humidity, 55%, in standard cages with a 12 h-12 h light-dark cycle. All animals had *ad libitum* access to water and a sterilized chow diet (Harlan diet 2018S, Koatech, Pyeongtaek, Gyeonggi-do, Korea). All experimental procedures were approved by the Korea University Institutional Animal Care and Use Committee (Approval number: KUIACUC-2017-32) and were in accordance with the Guide for the Care and Use of Laboratory Animals (NIH Publication No. 85–23, 1996). The experimental design used for animal study is shown in Supplementary Fig. S1. The rats were given a seven-day adaptation period and were then randomly divided into five groups (n = 6) and orally administered LPF (1 × 10^8^ CFU) or SPFC (1 mL) every day for 22 days. To induce acute colitis, the rats were given 5% DSS in their drinking water for the final 8 days of the experiment^16^. The rats in Group I (the negative control [NC]) were provided normal drinking water and were orally administered 1 mL PBS. The rats in Group II (the model group) were fed and treated in the same manner as group I, except they were supplied with 5% DSS in their drinking water for the last 8 days of the experiment to induce acute colitis. The rats in Group III (the LPF group), group IV (the RCM group), and group V (the SPFC group) were orally administered live *P. freudenreichii* (1 × 10^8^ CFU), RCM (1 mL), and SPFC (1 mL), respectively, from the day (8^th^ day) after adaptation to the end of animal experiment, and these groups were provided water with 5% DSS for the last 8 days of the experiment. The faeces of all of the rats were collected and stored at −80 °C until use.

### Histological analysis and IHC

The middle sections of the distal colons of each rat were fixed in 4% neutral buffered formalin (NBF) and embedded in paraffin, and 3 μm sections were obtained. The slides were stained with H&E or AB. To estimate colonic damage, the slides stained with H&E were observed by a phase contrast microscope (LEICA ICC50, Wetzlar, Germany) and scored according to the parameters shown in Supplementary Table S1^45^. Goblet cells in crypts were counted with the AB-stained specimens. For IHC, the antigen was retrieved using Tris-EDTA buffer (pH 9.0) and the detection procedure was performed using an UltraVision LP Large Volume Detection System: HRP Polymer (Thermo Scientific) according to the manufacturer’s instructions. The primary antibody against MUC2 (sc-7314, Santa Cruz, Dallas, TX, USA) was used, and the images obtained from the phase contrast microscope (LEICA ICC50) were analysed with ImageJ.

### Evaluation of the disease activity index (DAI) and colon length

To evaluate the DAI, the loss of body weight, consistency of faecal pellets, and signs of rectal bleeding were monitored in the rats while they were provided 5% DSS. The DAI scoring system was set up with the parameters described in Supplementary Table S2 with a minor modifications^46^. The scoring of body weight loss (%) was performed as follows: [(Body weight of each day after the rats were provided with 5% DSS)/(Body weight just before the rats were provided with 5% DSS)] × 100. The DAI value was calculated by summing the scores of body weight loss, stool consistency, and rectal bleeding; the total score ranged from 0 (normal) to 12 (severe colitis).

Colon of each rat on the last day of the experiment was collected, and measured for the length (cm) before maintaining the samples at −80 °C for use in further analyses.

### Measurement of gene expression by qPCR

Total RNA of each sample was extracted using 1 mL of Trizol reagent (Invitrogen, Carlsbad, CA, USA) following the manufacturer’s instructions. Total RNA was quantified with a NanoDrop ND-1000 spectrophotometer (Thermo Scientific, Wilmington, DE, USA) and converted to cDNA with a RevertAid First Strand cDNA Synthesis kit (Thermo Fisher Scientific). qPCR was done using a Kapa SYBR Fast qPCR kit (Kapa Biosystems, Woburn, MA, USA) with a StepOnePlus™ Real-Time PCR System (Applied Biosystems, Foster City, CA, USA). The PCR primer sequences are shown in Supplementary Table S3. The qPCR sample was preheated to 95 °C for 10 min followed by 40 cycles of 95 °C for 15 s, 60 °C for 15 s, and 72 °C for 20 s. *GAPDH* was used as the internal control gene. The data were analysed based on the 2^−ΔΔCt^ method (the *GAPDH* control was set to 1)^47^. For the animal samples, the residual DSS in the extracted total RNA from the colon specimens was removed with 8 M LiCl to prevent DSS from possibly being carried over from the colon specimens of the DSS-treated rats^48^, because DSS remaining in the colon specimens might inhibit the activity of reverse transcriptase. The total RNA was quantified and converted to cDNA with the same procedure described above except *β-actin* was used as the internal control gene.

### HPLC

To analyse the contents of SCFAs in the faeces of the rats, the faeces were dried, suspended in HPLC-graded water with a 1:3 dilution ratio (w:v), and centrifuged at 12,000 × *g* for 10 min at 4 °C. The supernatant was filtered with a 0.45 μm-pore size syringe filter (ADVANTEC, Tokyo, Japan). Acetate, propionate, and butyrate served as the standards for calibration, and their concentrations in each sample were detected using an UltiMate 3000 Rapid Separation LC System (Thermo Fisher Scientific, Waltham, MA, USA). An Aminex® HPX-87H ion exclusion column (300 mm × 7.8 mm, Bio-Rad, Hercules, CA, USA) was used at 55 °C. Filtered and degassed 0.005 M H_2_SO_4_ was used as the mobile phase in an isocratic manner, and the flow rate was 0.6 mL/min. The total running time was 50 min, and the SCFAs were detected at a wavelength of 210 nm. The concentrations of SCFAs in each sample were expressed as the mean μmol/g faeces. The HPLC profiles for acetate, propionate, and butyrate are shown in Supplementary Fig. S5.

### *Propionibacterium*-selective media

To estimate whether *P. freudenreichii* was established in the gut, YELA medium containing 1% tryptone, 1% yeast extract, 0.025% K_2_HPO_4_, 0.005% MnSO_4_, 1% sodium lactate, and 1.5% agar (pH 7.0 ± 0.05) was used to enumerate the amount of *Propionibacterium* in the faeces^49^. The faeces of the LPF group on days 7, 14, 21, and 29 were collected and suspended in PBS at a 1:3 dilution ratio. Subsequently, the samples were spread onto YELA medium and incubated at 30 °C for 6 days in an anaerobic chamber with a GasPak (Becton Dickinson, Franklin Lakes, NJ, USA). Brown colonies (≥1 mm in diameter) were enumerated as *Propionibacterium* colonies on YELA.

### Statistical analysis

All statistical analyses were performed using the Statistical Package for the Social Sciences (SPSS, Chicago, IL, USA) software package version 24.0. Data are expressed as the mean ± standard deviation (SD) of three independent experiments performed in triplicate *in vitro* and the mean ± SD of the experiments performed *in vivo*. The significance of the differences was determined by the Student’s *t*-test for the *in vitro* experiments. The significance of the differences between groups was determined by Mann-Whitney test. A *P* value of <0.05 was considered significant.

## Supplementary information

Supplementary Information.
