# Supplementary material for: Dairy Propionibacterium freudenreichii ameliorates acute colitis by stimulating MUC2 expression in intestinal goblet cell in a DSS-induced colitis rat model
Source: Sci Rep. 2020 Mar 26;10:5523. doi: 10.1038/s41598-020-62497-8 (PMC7099060; doi:10.1038/s41598-020-62497-8)
Supplement: Supplementary file 1 — Supplementary Information. [file 41598_2020_62497_MOESM1_ESM.pdf]

Supplementary information

Dairy *Propionibacterium freudenreichii* ameliorates acute colitis by stimulating MUC2 expression in intestinal goblet cell in a DSS-induced colitis rat model

Seongho Ma, Jiah Yeom & Young-Hee Lim

Supplementary Table S1. Parameters for estimating histology.

Score	Surface epithelial Loss	Crypt Destruction	Inflammatory cell infiltration
0	Normal	Normal	Normal
1	Localized & mild	Localized & mild	Localized & mild
2	Localized & moderate	Localized & moderate	Localized & moderate
3	Extensive & moderate	Extensive & moderate	Extensive & moderate
4	Extensive & severe	Extensive & severe	Extensive & severe

Supplementary Table S2. Parameters for evaluating disease activity index (DAI).

Score	Body weight loss	Stool consistency	Bleeding
0	None	Normal	None
1	1 – 5%	Soft but maintain morphology	A little bloodstain
2	5 – 10%	Soft	Apparent bloodstain on fecal pellets & rectum
3	10 – 20%	Very soft	
4	> 20%	Diarrhea	Gross bleeding

Supplementary Table S3. Primers used for qPCR analysis *in vitro* and *in vivo*.

Gene	Forward (5' to 3')	Reverse (5' to 3')
human *GAPDH*	GAG TCA ACG GAT TTG GTC GT	TTG ATT TTG GAG GGA TCT CG
human *MUC2*	ACC CGC ACT ATG TCA CCT TC	GGA CAG GAC ACC TTG TCG TT
rat *β-actin*	AGC CAT GTA CGT AGC CAT CC	CTC TCA GCT GTG GTG GTG AA
rat *MUC2*	CCA TCA CGG AGA CTT CCA CT	CTG TGG TGG TGG AGA CTG TG
rat *TNF-α*	ATG TGG AAC TGG CAG AGG AG	GGC CAT GGA ACT GAT GAG AG
rat *IL-6*	CCG GAG AGG AGA CTT CAC AG	ACA GTG CAT CAT CGC TGT TC
rat *IL-1β*	AGG CAG TGT CAC TCA TTG TG	GGA GAG CTT TCA GCT CAC AT
rat *IL-10*	GCT CAG CAC TGC TAT GTT GC	TGT CCA GCT GGT CCT TCT TT

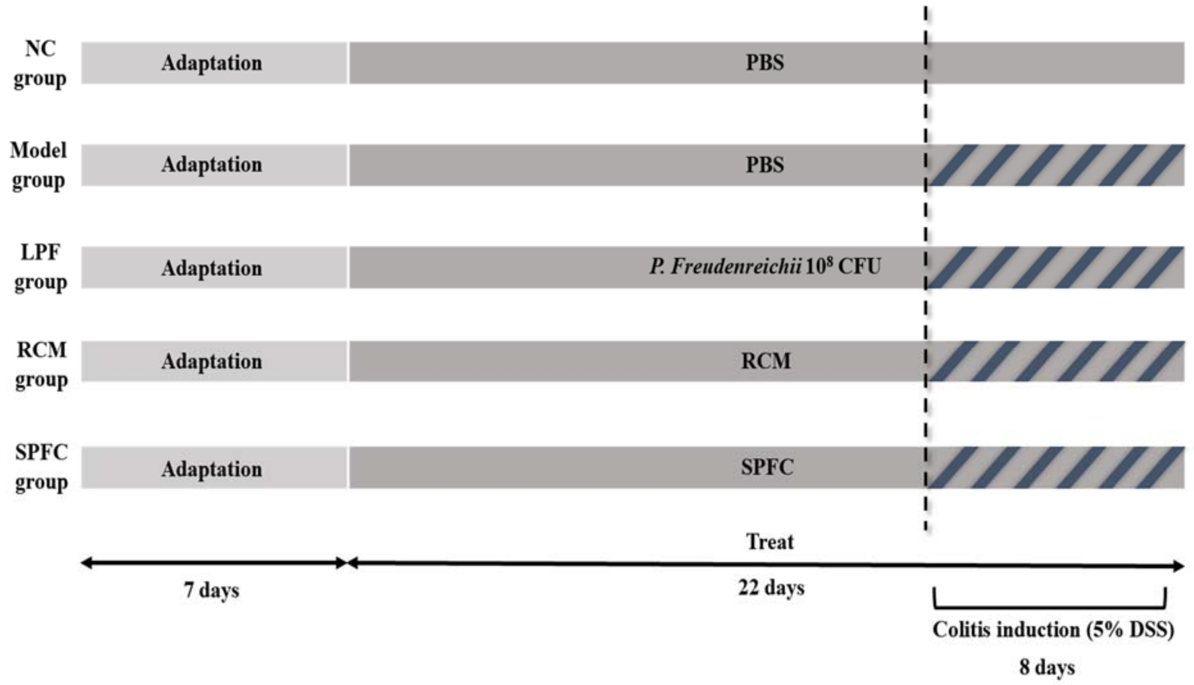

Supplementary Fig. S1. Animal study design.

After all rats were given a week for adaptation, Phosphate buffered saline (PBS) was orally administered to the negative control (NC) and model group. LPF, RCM, and SPFC groups were treated with live *P. freudenreichii* (10^8 CFU), Reinforced Clostridial Medium (RCM) 1 mL, and supernatant of *P. freudenreichii* culture (SPFC) 1 mL, respectively. For the last eight days, all groups except the negative control were co-treated with 5% dextran sodium sulfate (DSS) to induce acute colitis. RCM The model (DSS only-treated group) and RCM groups were served as experimental controls for the LPF and SPFC groups, respectively.

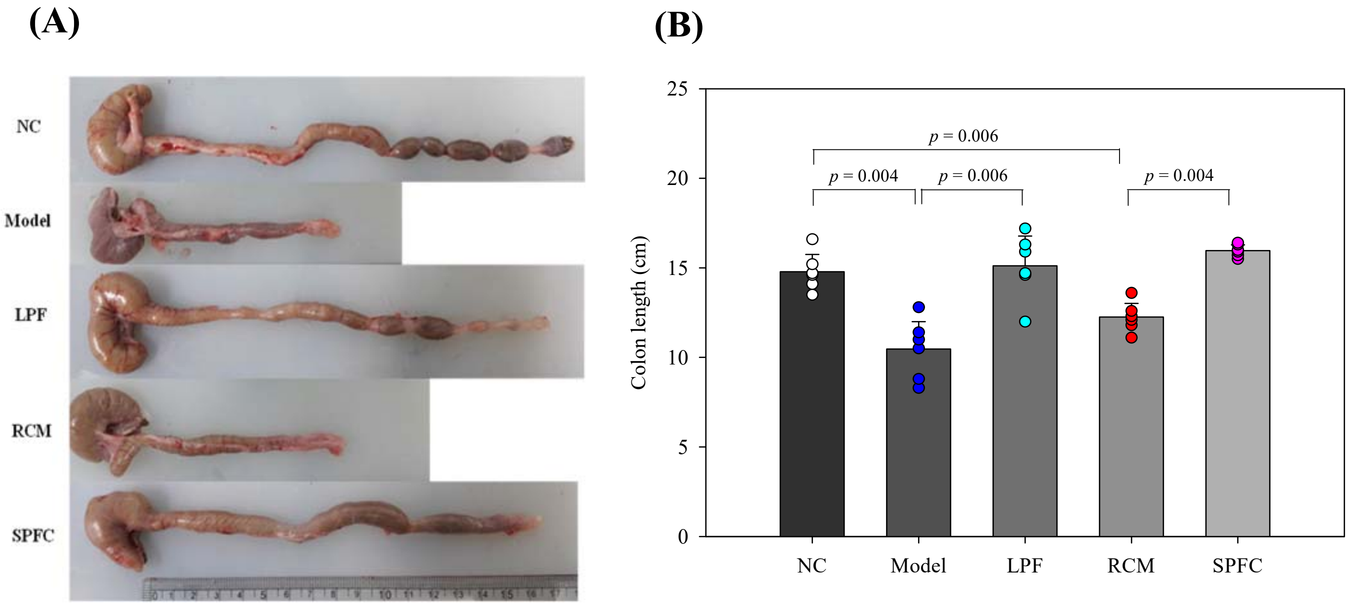

Supplementary Fig. S2. Effects of LPF and SPFC on colon length in DSS-induced acute colitis rats.

Colons were isolated and measured for their length (A and B). Data are expressed as mean ± SD (n = 6).

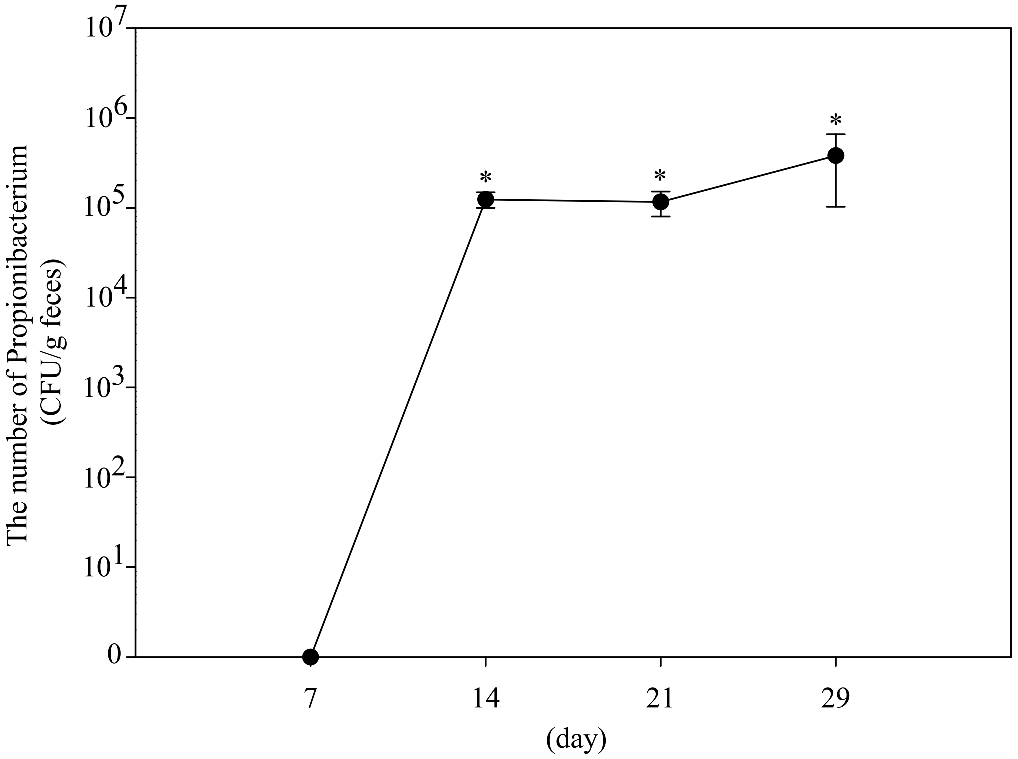

Supplementary Fig. S3. The number of live *Propionibacterium* in feces of the LPF group.

Live *Propionibacterium* in feces of LPF group was counted with fecal samples on day 7, 14, 21, and 29 to analyze its settlement on the gut during the term of administration of LPF for 3 weeks. The 4-week sample was taken just after 5% DSS treatment with LPF. The data are expressed as mean ± SD (CFU/g feces) (n = 6). *$p < 0.05$, Student's *t*-test, compared with the 1-week, the last day of adaptation.

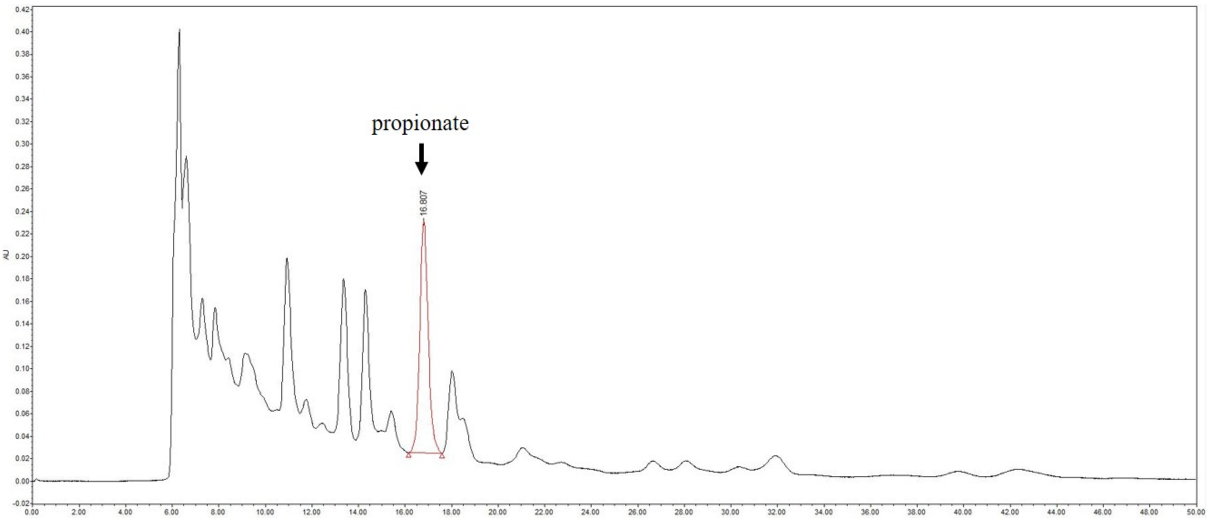

Supplementary Fig. S4. A HPLC graph of propionate in SPFC.

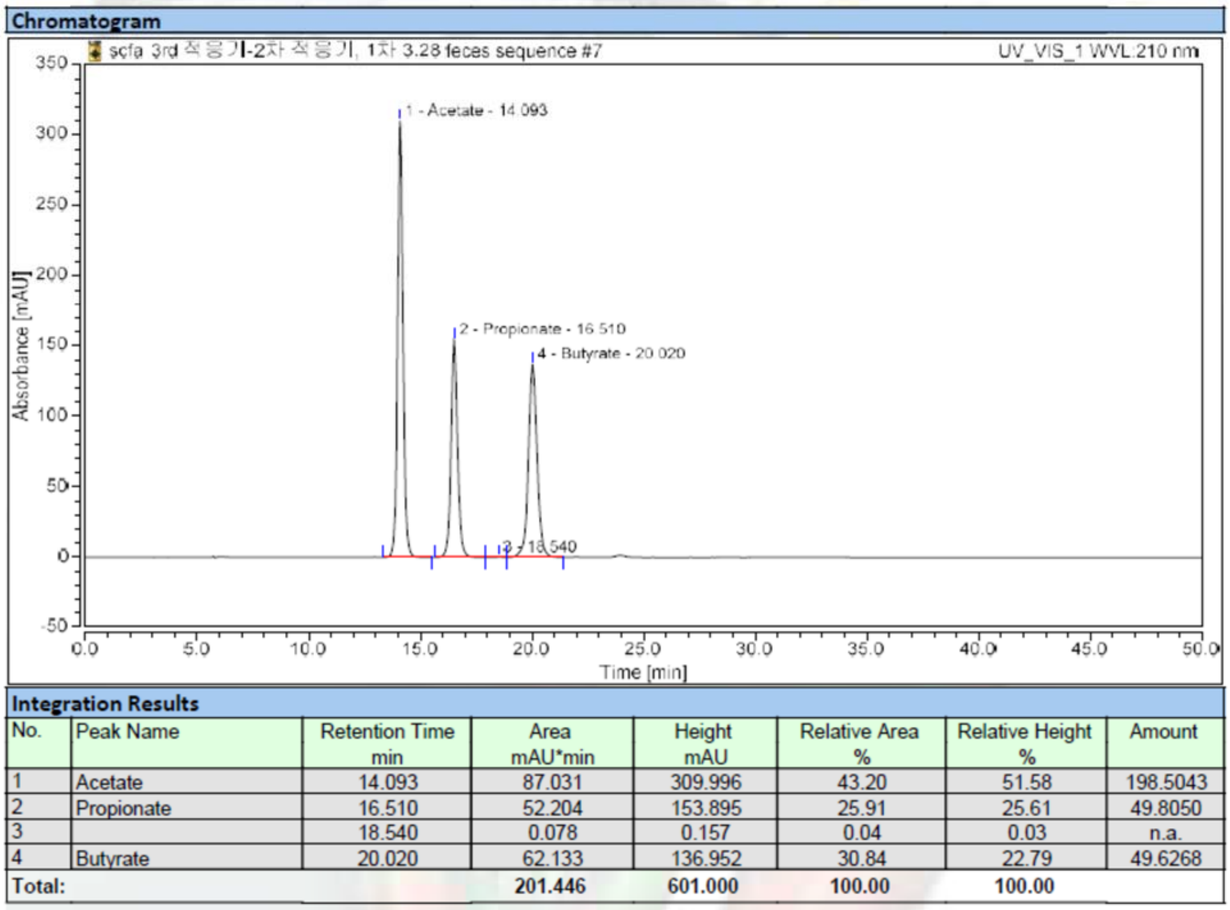

Integration Results

No.	Peak Name	Retention Time min	Area mAU*min	Height mAU	Relative Area %	Relative Height %	Amount
1	Acetate	14.093	87.031	309.996	43.20	51.58	198.5043
2	Propionate	16.510	52.204	153.895	25.91	25.61	49.8050
3		18.540	0.078	0.157	0.04	0.03	n.a.
4	Butyrate	20.020	62.133	136.952	30.84	22.79	49.6268
**Total:**			**201.446**	**601.000**	**100.00**	**100.00**	

Supplementary Fig. S5. A HPLC profile of acetate, propionate, and butyrate.
